# Combining D-dimer and LDL/HDL ratio to predict the absence of atrial fibrillation in patients with an Implantable Loop Recorder for embolic stroke of Undetermined source

**DOI:** 10.1016/j.ijcha.2025.101611

**Published:** 2025-01-16

**Authors:** Kosuke Yoshikawa, Taku Asano, Yoshimi Onishi, Yuki Takai, Hiroto Sugiyama, Shuhei Arai, Toshihiko Gokan, Yuya Nakamura, Koichiro Inokuchi, Miwa Kikuchi, Tatsuya Onuki, Hitoshi Ezumi, Shinji Koba, Kaoru Tanno, Youichi Kobayashi, Toshiro Shinke

**Affiliations:** a Division of Cardiology, Department of Medicine, Showa University, Tokyo, Japan; b Division of Cardiology, Department of Medicine, Showa University Fujigaoka Hospital, Yokohama, Japan; c Cardiovascular Center, Showa University Koto Toyosu Hospital, Tokyo, Japan; d Division of Cardiology, Koyama Memorial Hospital, Ibaraki, Japan

**Keywords:** Atrial Fibrillation, Implantable Loop Recorder, Embolic Stroke of Undetermined Source, D-dimer, LDL/HDL cholesterol ratio

## Abstract

**Background and Objective:**

Embolic stroke of undetermined source (ESUS) patients undergoing long-term rhythm monitoring with Implantable Loop Recorder (ILR) have an atrial fibrillation (AF) detection rate of approximately 12 % at 1 year and 30 % with extended follow-up over 3 years. However, research specifically focusing on the majority of patients in whom AF is not detected through implantable cardiac monitors remains limited. Abnormal lipid profiles may be associated with embolic risks from non-AF sources. This study aimed to develop a model to predict the absence of AF in patients with ESUS using multiple variables, including lipid profiles.

**Methods:**

A retrospective, multicenter cohort study was conducted across four institutions, involving 99 ESUS patients. Patients were categorized based on AF detection via ILR. Patient characteristics, blood test results, and echocardiographic findings were assessed through univariate and multivariate logistic regression analyses. ROC curve analysis was performed to evaluate the biomarkers’ predictive accuracy.

**Results:**

AF was detected in 30.3 % of patients over a median follow-up of 25.5 months. Multivariate analysis confirmed elevated D-dimer (OR: 2.77, p = 0.002), low LDL/HDL ratio (OR: 2.0, p = 0.01), and CHA2DS2-VASc score (OR: 1.4, p = 0.04) as independent predictors of AF detection. The CHA2DS2-VASc score was excluded due to multicollinearity, and patients with D-dimer < 0.9 μg/ml and LDL/HDL ratio > 1.98 had significantly lower AF detection rates (6.8 %, P < 0.001; sensitivity 93.1 %, specificity 44.2 %).

**Conclusion:**

Combining D-dimer and LDL/HDL ratios provides an effective and accessible method for predicting the absence of AF in patients with an ILR for ESUS.

## Graphical abstract

**Figure:**
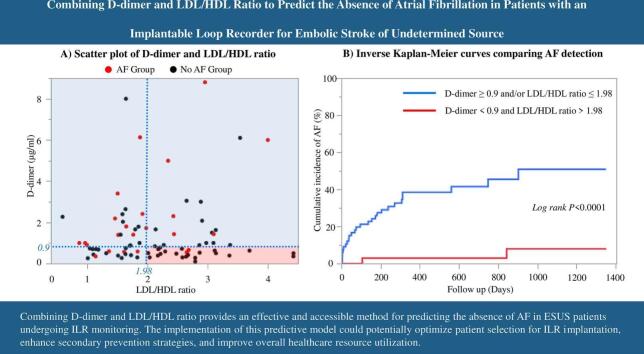

## 1 Introduction

Stroke is a major cause of morbidity and mortality worldwide, making the identification of its etiology critical for optimizing secondary prevention strategies. Despite advances in diagnostic techniques, 20–30 % of strokes remain unexplained and are often classified as cryptogenic. [1] To address this challenge, the concept of Embolic Stroke of Undetermined Source (ESUS) was introduced in 2014. ESUS is characterized as a non-lacunar ischemic stroke without proximal arterial stenosis or a confirmed cardiac source of embolism, suggesting the presence of an undetected embolic source [1].

Implantable loop recorders (ILR) are utilized to detect subclinical atrial fibrillation (AF) in ESUS patients, playing a critical role in identifying potential causes of embolic strokes. The definitive diagnosis of AF through ILR facilitates targeted secondary prevention, potentially improving patient outcomes. However, ESUS patients undergoing long-term rhythm monitoring with ILR have an AF detection rate of approximately 12 % at 1 year and 30 % with extended follow-up over 3 years [2].

Previous studies have identified several predictors of AF detection in ESUS patients using ILR, including age, B-type natriuretic peptide (BNP) levels, left atrial diameter, left ventricular diastolic dysfunction, premature atrial contractions on Holter electrocardiogram (ECG), and CHA2DS2-VASc score. [3], [4], [5], [6] However, research focused on abnormal findings in ESUS patients without detected AF is limited. A recent study by Komatsu et al. reported that aortic arch plaque thickening was observed in ESUS patients without detected AF, highlighting specific risk factors pertinent to this subgroup [7].

Carotid artery plaques are well-documented to be associated with abnormalities in lipid metabolism. [8] Therefore, dyslipidemia may represent a significant risk factor for ESUS caused by mechanisms other than AF. Although Vitturi et al. recently demonstrated that statins effectively reduce ischemic stroke recurrence in ESUS patients, their findings also highlight the potential role of unfavorable lipid profiles as a major contributor to ESUS. However, the role of these lipid profiles in AF-negative ESUS remains insufficiently studied. [9] Therefore, we aimed to investigate whether dyslipidemic profiles can serve as predictive markers for the absence of AF in ESUS patients, using dyslipidemic profiles as key variables. Furthermore, we assessed whether combining these lipid markers with established AF detection predictors, including D-dimer—an indicator of intracardiac thrombus formation—could enhance the model's accuracy in identifying patients unlikely to benefit from ILR implantation. To account for potential significant but unforeseen risk factors, demographic characteristics, clinical features, and echocardiographic parameters were included in the analysis.

## 2 Methods

### 2.1 Study design

This study was a retrospective, multicenter cohort investigation conducted across four institutions: Showa University Hospital, Showa University Koto Toyosu Hospital, Showa University Fujigaoka Hospital, and Koyama Memorial Hospital.

### 2.2 Inclusion criteria

Patients with an MRI-confirmed diagnosis of ischemic stroke classified as ESUS, based on a comprehensive diagnostic evaluation, were included in the analysis.

### 2.3 Exclusion criteria

Exclusion criteria were: patients who have not undergone a minimum follow-up of 6 months, significant impairments in activities of daily living precluding reliable follow-up, and refusal to participate in the study. Patients with known malignant diseases were excluded from the study to avoid confounding factors related to cancer-associated hypercoagulability.

### 2.4 ESUS diagnostic criteria

Stroke subtype was determined using the ESUS consensus criteria [1] by the treating vascular neurologist and adjudicated independently by another vascular neurologist, and, in case of disagreement, a discussion was held between the two and a consensus was achieved. Patients underwent extensive diagnostic testing, including blood tests, 12-lead ECG, 24-hour Holter monitoring, transthoracic echocardiography, head and neck computed tomography/magnetic resonance imaging (CT/MRI), carotid ultrasound, lower limb venous ultrasound, and transesophageal echocardiography to exclude other stroke etiologies. ESUS was defined based on the following criteria:

1. Non-lacunar ischemic stroke or transient ischemic attack confirmed by neuroimaging;
2. Absence of stenosis or occlusion greater than 50 % in arteries supplying the ischemic area;
3. No recorded AF on 24-hour Holter ECG;
4. Absence of intracardiac thrombus on transthoracic echocardiography;
5. Absence of other major cardioembolic sources;
6. No other identified cause of stroke (e.g., arteritis, dissection, or vasospasm).

All patients underwent a 24-hour Holter ECG within one week of hospitalization, ensuring comprehensive monitoring before ILR implantation. Continuous monitoring was conducted up to the time of ILR implantation.

### 2.5 Patient Classification

Patients were classified into two groups: those in whom AF lasting more than 6 min was detected (AF Group) and those in whom AF lasting 6 min or less was detected, or no AF was detected (No AF Group), based on ILR monitoring.

### 2.6 Follow-up

Patients with ESUS and ILR were followed up every 3–6 months to detect the onset of AF. During each visit, data from the ILR were reviewed, and any detected AF episodes were validated using Medtronic's AF detection algorithm. Each episode was confirmed by a cardiologist. Notably, patients were not continuously monitored remotely during the follow-up period. Demographic data, clinical test results, and the presence of AF during follow-up were analyzed to identify predictive factors. All patients received a Medtronic Reveal LINQ® ILR and were followed for a minimum of six months.

### 2.7 Comparative data

Demographic characteristics (age, sex, height, weight, and BMI), blood test results (including lipid profiles such as triglycerides, HDL cholesterol, LDL cholesterol, and LDL/HDL ratio, as well as D-dimer and B-type natriuretic peptide), echocardiographic findings (left atrial diameter, left atrial volume, and ejection fraction), and clinical risk scores (CHA2DS2-VASc, HAVOC, and Brown ESUS-AF scores) were compared between the AF Group and No AF Group to identify significant differences and potential predictors of AF detection.

### 2.8 HAVOC, Brown ESUS-AF, and CHA2DS2-VASc scores

All patients were assessed using the HAVOC, Brown ESUS-AF, and CHA2DS2-VASc scores. The HAVOC score was calculated by assigning points for hypertension (2 points), age ≥ 75 years (2 points), valvular heart disease (2 points), peripheral vascular disease (1 point), obesity (BMI > 30 kg/m^2^) (1 point), heart failure (4 points), and coronary artery disease (2 points), with a total possible score ranging from 0 to 14. [10] The Brown ESUS-AF score was determined based on age (65–74 years: 1 point; ≥75 years: 2 points) and moderate/severe left atrial enlargement (LA volume index > 34 ml/m^2^: 2 points), with scores ranging from 0 to 4 [11].

### 2.9 Statistical analysis

Continuous variables were expressed as mean ± standard deviation, and categorical variables as frequencies and percentages. Continuous variables were compared using the Mann-Whitney *U* test, while categorical variables were compared using the Chi-square test. Cox proportional hazards regression analysis was conducted to identify significant independent predictors of AF detection, with odds ratios (OR) and 95 % confidence intervals (CI) calculated. Receiver operating characteristic (ROC) curve analysis was performed to evaluate the predictive accuracy of variables for AF detection. Kaplan-Meier curves were utilized to analyze the time from ILR implantation to AF detection. A p-value < 0.05 was considered statistically significant. Statistical analyses were conducted using JMP software version 16.0.

### 2.10 Standard protocol Approvals and Registrations

This study protocol was approved by the Ethics Committee of Showa University, Japan, and was conducted in accordance with the principles of the Declaration of Helsinki (Approval Number: 3138). The requirement for informed consent was waived due to the retrospective nature of the study and the minimal risk to participants.

## 3 Results

### 3.1 Follow-up and AF detection

Between September 2016 and March 2021, 99 ESUS patients admitted to the four hospitals were enrolled, and their follow-up data were analyzed. The median time from ESUS onset to ILR implantation was 25.5 ± 18.8 days. The median follow-up duration was 25.5 ± 12.8 months (interquartile range, 13–38 months). During follow-up, AF was detected in 30 out of 99 patients (30.3 %), all of whom were asymptomatic. The median time to AF detection via ILR was 105 days (interquartile range 29–272 days). Notably, two patients who initially exhibited AF episodes shorter than 6 min subsequently developed episodes exceeding 6 min during follow-up. In all ESUS patients, antiplatelet therapy was initiated following diagnosis, as anticoagulation was not indicated at the time. After the detection of AF, patients were switched from antiplatelet agents to anticoagulants. During the follow-up period, no recurrent strokes, including recurrent ESUS events, were observed in any of the patients.

### 3.2 Patient characteristics (Table 1)

#### 3.2.1 Demographic and Baseline characteristics

The mean age of the patients was 66.2 ± 13.1 years, with 65.7 % being male. Hypertension was the most common comorbidity, present in 65.7 % of patients. The AF Group was significantly older (73.9 ± 7.5 years vs. 62.9 ± 13.7 years, P = 0.0002) than the No AF Group. Males were more prevalent in the No AF Group, leading to greater average height and weight, although no significant difference in BMI was observed. There was no significant difference in statin usage between the AF Group and No AF Group Table 1.

**Table 1:** Patient characteristics.

	Baseline Characteristics			
	Total (N = 99)	AF Group (N = 30)	No AF Group (N = 69)	P-value
Patient characteristics				
**Male**	65 (65.7 %)	16 (53.3 %)	49 (65.7 %)	0.09
**Age**	66.2 ± 13.1	73.9 ± 7.5	62.9 ± 13.7	0.0002
**Height (cm)**	161.7 ± 9.8	158.5 ± 11.1	163.1 ± 8.9	0.047
**Weight (kg)**	61.6 ± 12.7	58.2 ± 10.1	63.1 ± 13.5	0.028
**BMI (kg/m^2^)**	23.4 ± 3.9	23.3 ± 3.8	23.4 ± 4.0	0.6
**Smoking**	57 (60.6 %)	13 (46.4 %)	44 (66.7 %)	0.06
**Drinking alcohol**	34 (36.2 %)	9 (32.1 %)	25 (37.9 %)	0.6
**HT**	65 (65.7 %)	20 (66.7 %)	45 (65.2 %)	0.8
**DM**	26 (26.5 %)	7 (23.3 %)	19 (27.9 %)	0.6
**DL**	58 (59.2 %)	16 (53.3 %)	42 (61.8 %)	0.4
**CKD**	10 (10.2 %)	6 (20 %)	4 (5.9 %)	0.04
**IHD**	9 (9.1 %)	3 (10 %)	6 (8.7 %)	0.4
LDL cholesterol-lowering therapy				
**Statin**	17 (17.2 %)	4 (13.3 %)	13 (18.8 %)	0.6
Echocardiography Findings				
**EF (%)**	64.1 ± 10.1	65 ± 10.5	63.8 ± 9.9	0.7
**LAD (mm)**	35.4 ± 6.7	38.7 ± 7.2	34.0 ± 5.9	0.002
**LA volume (ml)**	40.9 ± 20.5	52.1 ± 26.2	34.8 ± 13.6	0.004
**RVSP (mmHg)**	25.5 ± 8.2	25.5 ± 9.8	25.5 ± 7.3	0.7
Biochemical Findings				
**Hb (g/dl)**	13.9 ± 1.9	13 ± 1.6	14.1 ± 2.0	0.04
**HDL cholesterol (mg/dl)**	58.4 ± 16.2	65.2 ± 18.3	55.3 ± 14.3	0.01
**LDL cholesterol (mg/dl)**	121.4 ± 38.2	115.0 ± 33.4	124.2 ± 40.0	0.3
**LDL/HDL ratio**	2.6 ± 1.1	1.9 ± 0.9	2.9 ± 1.1	0.01
**TG (mg/dl)**	137.6 ± 96.8	98.4 ± 47.8	155.4 ± 100.7	0.007
**BNP (pg/ml)**	73.9 ± 144.9	143.3 ± 217.3	38.4 ± 67.4	0.0001
**D-dimer (μg/ml)**	2.5 ± 0.3	2.8 ± 3.6	1.3 ± 1.8	0.001
Diagnostic testing				
**Blood exam**	99 (100 %)	30 (100 %)	69 (100 %)	1.0
**ECG**	99 (100 %)	30 (100 %)	69 (100 %)	1.0
**Brain CT**	99 (100 %)	30 (100 %)	69 (100 %)	1.0
**Brain MRI**	99 (100 %)	30 (100 %)	69 (100 %)	1.0
**UCG**	97 (98.0 %)	29 (96.7 %)	68 (98.6 %)	0.2
**TEE**	50 (50.5 %)	18 (60.0 %)	32 (46.4 %)	0.1
**Carotid US**	80 (80.8 %)	22 (73.3 %)	58 (84.1 %)	0.4
**LVUS**	64 (64.7 %)	20 (66.7 %)	44 (63.8 %)	0.6
**Holter ECG**	67 (67.7 %)	22 (73.3 %)	45 (65.2 %)	0.3
AF risk score				
**HAVOC**	3.1 ± 2.5	4.7 ± 2.9	2.5 ± 2.0	<0.001
**Brown ESUS-AF**	1.2 ± 1.2	2.2 ± 1.3	0.8 ± 0.9	<0.001
**CHA2DS2-VASc**	4.3 ± 1.4	5.0 ± 1.3	4.0 ± 1.3	0.002

BMI: Body Mass Index. HT: Hypertension. DM: Diabetes mellitus. DL: Dyslipidemia. CKD: Chronic kidney disease. IHD: Ischemic heart disease. LDL: Low Density Lipoprotein. EF: Ejection fraction. LAD: Left atrial dimension. LA volume: Left atrial volume. RVSP: Right ventricular systolic pressure. Hb: Hemoglobin. HDL: High Density Lipoprotein. TG: Triglyceride. BNP: Brain natriuretic peptide. ECG: electrocardiography. CT: computed tomography. MRI: magnetic resonance imaging. UCG: ultrasound cardiography. TEE: transesophageal echocardiography. US: ultrasound. LVUS: lower limb venous ultrasound.

#### 3.2.2 Echocardiography findings

The AF Group had significantly larger left atrial diameters and volumes compared to the No AF Group (38.8 ± 7.2 mm vs. 34.0 ± 5.9 mm, P = 0.002; 52.1 ± 26.2 ml vs. 34.8 ± 13.6 ml, P = 0.004). No significant differences were observed in ejection fraction between the groups (65 ± 10.5 % vs. 63.8 ± 9.9 %, P = 0.7).

#### 3.2.3 Biochemical findings

The AF Group exhibited significantly elevated levels of D-dimer (2.8 ± 3.6 μg/ml vs. 1.3 ± 1.7 μg/ml, P = 0.001) and BNP (143.3 ± 217.3 pg/ml vs. 38.4 ± 67.4 pg/ml, P < 0.001). Conversely, the No AF Group had lower high-density lipoprotein (HDL) cholesterol levels (65.2 ± 18.3 mg/dl vs. 55.3 ± 14.3 mg/dl, P = 0.01), a higher low-density lipoprotein to high-density lipoprotein cholesterol ratio (LDL/HDL ratio) (1.9 ± 0.9 vs. 2.9 ± 1.1, P = 0.006), and higher triglyceride (TG) levels (98.4 ± 47.8 vs. 155.4 ± 107.8, P = 0.007).

#### 3.2.4 HAVOC, Brown ESUS-AF, and CHA2DS2-VASc scores

HAVOC score (4.7 ± 2.9 vs. 2.5 ± 2.0, P < 0.001), Brown ESUS-AF score (2.2 ± 1.3 vs. 0.8 ± 0.9, P < 0.001), and The CHA2DS2-VASc score (5.0 ± 1.3 vs. 4.0 ± 1.3, P = 0.002) showed significant differences between the AF Group and No AF Group.

### 3.3 Univariate and multivariate analysis (Table 2)

Univariate analysis identified several factors as significant predictors of AF detection, including height, age, left atrial diameter, left atrial volume, D-dimer, BNP, TG, chronic kidney disease, hemoglobin, HDL cholesterol, and LDL/HDL ratio. Additionally, CHA2DS2-VASc score, HAVOC score, and Brown ESUS-AF Score were also significant predictors of AF detection. In multivariate analysis, three independent predictors of AF detection were identified: elevated D-dimer (OR: 2.77, 95 % CI: 1.19–9.87, p = 0.001), a low LDL/HDL ratio (OR: 1.97, 95 % CI: 1.11–6.51, p = 0.01), and CHA2DS2-VASc score (OR: 1.37, 95 % CI: 1.05–4.10, p = 0.04).　However, the CHA2DS2-VASc score was excluded from the final predictive model due to multicollinearity with other variables, which could compromise the model's statistical robustness and interpretability Table 2.

**Table 2:** Result of univariable and multivariable analysis for AF detection.

	Univariable analysis		Multivariable analysis	
	OR(95 % CI)	P value	OR(95 % CI)	P value
**Age**	1.08(1.03–1.13)	<0.001		
**Height**	0.95(0.91–0.99)	0.03		
**LAD**	1.12(1.05–1.22)	<0.001		
**LA volume**	1.04(1.02–1.08)	<0.001		
**HDL cholesterol**	1.03(1.01–1.07)	0.01		
**LDL/HDL ratio**	0.51(0.28–0.90)	0.01	1.97(1.11–6.51)	0.01
**TG**	0.98(0.97–0.99)	0.001		
**BNP**	1.01(1.00–1.02)	<0.001		
**D-dimer**	1.28(1.03–1.61)	0.01	2.77(1.19–9.87)	0.001
**HAVOC**	1.46(1.20–1.79)	<0.001		
**Brown ESUS-AF**	2.95(1.81–4.78)	<0.001		
**CHA2DS2-VASc**	1.79(1.23–2.59)	<0.001	1.37(1.05–4.10)	0.04

Abbreviations as in Table 1.

### 3.4 Predictive value of combining D-dimer and LDL/HDL ratio for AF detection

**Fig. 1:**
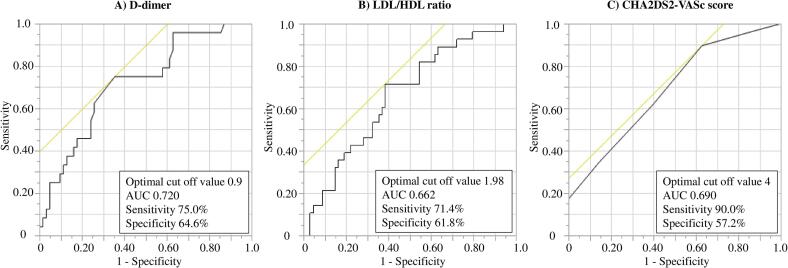
ROC curves for AF detection based on individual predictors. (A) D-dimer: The ROC curve for D-dimer demonstrated an AUC of 0.72, indicating moderate predictive accuracy. (B) LDL/HDL ratio: The ROC curve for LDL/HDL ratio displayed an AUC of 0.66, reflecting fair predictive accuracy. (C) CHA2DS2-VASc score: The ROC curve for the CHA2DS2-VASc score presented an AUC of 0.69, suggesting moderate predictive accuracy. Other Abbreviations as in Table 1.

The optimal cutoff values for D-dimer and LDL/HDL ratio were determined to be 0.9 μg/ml and 1.98, respectively. For D-dimer, the sensitivity was 75.0 %, specificity was 64.6 %, and the area under the curve (AUC) was 0.720. For LDL/HDL ratio, the sensitivity was 71.4 %, specificity was 61.8 %, and the AUC was 0.662. The optimal cutoff values for CHA2DS2-VASc score was determined to be 4 points. For CHA2DS2-VASc score, the sensitivity was 90.0 %, specificity was 57.2 %, and the AUC was 0.690. (Fig. 1).

**Fig. 2:**
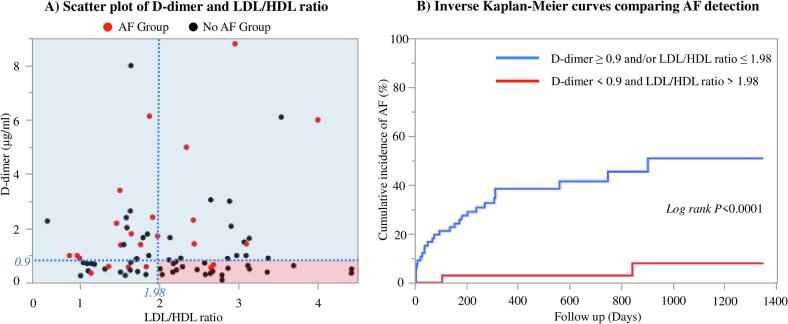
Scatter plot and Kaplan-Meier curve. (A) Scatter plot of D-dimer levels and LDL/HDL ratio, Patients with an LDL/HDL ratio > 1.98 and D-dimer < 0.9 μg/ml had a lower rate of AF detection (6.8 %, P < 0.001, sensitivity 93.1 %, specificity 44.2 %). (B) Inverse Kaplan-Meier curves comparing AF detection Kaplan-Meier curve comparing AF detection between patients with LDL/HDL ratio > 1.98 and D-dimer < 0.9 μg/ml and those who did not meet these criteria. A significant difference in AF detection rates was observed between the two groups (P < 0.0001). Abbreviations as in Table 1.

Among the 29 patients with D-dimer levels below 0.9 μg/ml and an LDL/HDL ratio above 1.98, AF was detected in only 2 patients (6.9 %), demonstrating a sensitivity of 93.1 % and a specificity of 44.2 % for the combined model. Kaplan-Meier analysis revealed a significant difference in AF detection rates between patients meeting these criteria and those who did not, with the log-rank test confirming this difference (P < 0.0001; Fig. 2).

## 4 Discussion

This study is the first to emphasize the clinical utility of combining D-dimer levels and the LDL/HDL ratio specifically for predicting the non-detection of AF in ESUS patients with ILR monitoring. This approach helps identify patients who are less likely to benefit from ILR use, optimizing resource allocation. The significant clinical value of these findings lies in the fact that both markers are derived from routine screening tests commonly conducted at the time of initial stroke evaluation, allowing for quick and easy risk stratification of ESUS patients.

The detection rate of AF in our study was 30.3 %, with a median detection time of 105 days, aligning with previous reports. [12], [13], [14], [15] The decision to define the AF Group based on episodes lasting over 6 min is consistent with criteria from large-scale clinical trials, further reinforcing the relevance of our findings [16], [17].

In the AF group, univariate analysis identified elevated D-dimer, BNP levels, increased left atrial diameter and volume, and advanced age as significant predictors of AF detection. Multivariate analysis confirmed that elevated D-dimer was independently associated with AF detection. Notably, with the exception of D-dimer, the other factors identified in univariate analysis have been previously reported in similar studies, [3], [4], [5], [6] further validating the robustness and consistency of our findings in the detection of AF in ESUS patients. The independent association of elevated D-dimer with AF suggests it may reflect ongoing fibrinolytic activity, possibly linked to subclinical thrombogenesis in the left atrium − a known source of emboli in AF-related strokes. [18], [19] Despite age-related influences on D-dimer levels, [20], [21], [22] our analysis confirmed its independent predictive value, underscoring its importance in assessing AF risk.

Conversely, the non-AF group displayed a lipid profile characterized by elevated TG, low HDL cholesterol, and a higher LDL/HDL ratio. Multivariate analysis demonstrated that a higher LDL/HDL ratio was independently associated with the absence of AF, suggesting its potential role in identifying non-AF ESUS patients. The higher LDL/HDL ratio's association with the absence of AF suggests that dyslipidemia may contribute to the development of atherosclerotic plaques, particularly in the aortic arch, potentially serving as alternative embolic sources in ESUS patients without AF. [23] Prior studies have linked lipid abnormalities with increased intima-media thickness, further supporting the role of dyslipidemia in non-AF embolic strokes. [24], [25], [26], [27].

The cost-effectiveness of ILR implantation for preventing recurrent stroke in ESUS patients has been evaluated in several studies across Europe and the United States, demonstrating variable results. [28], [29] The optimal cutoff values identified were D-dimer > 0.9 μg/ml and LDL/HDL ratio < 1.98. Patients who did not meet these criteria had a low AF detection rate of 6.9 %, suggesting that ILR implantation might not be necessary for these patients, which could enhance the cost-effectiveness of ILR use in the ESUS population.

The ESC guidelines recommend using HAVOC scores ≥ 4 or Brown ESUS-AF scores ≥ 2 to determine eligibility for ILR implantation after cryptogenic stroke. [30] However, these scores were developed based on patients identified through traditional, less sensitive methods for AF detection, without the use of ILR implantation. [9], [10] Therefore, these scores may have limitations in accurately predicting AF risk in ESUS patients. [31] In our study, although these scores were significant in univariate analysis for AF detection, they did not remain significant in multivariate analysis. On the other hand, the CHA2DS2-VASc score, although originally designed for stratifying stroke risk in AF patients, [CHA2DS2] has been reported as a predictor of AF in ESUS patients. [5] In our study, the CHA2DS2-VASc score was also a significant predictor of AF detection in multivariate analysis. However, the decision to exclude the CHA2DS2-VASc score from the final model was driven by multicollinearity concerns, allowing the focus to shift to simpler and clinically actionable predictors, such as LDL/HDL ratio and D-dimer levels. This approach aimed to enhance the model's practical utility in identifying patients at low risk of AF.

Currently, there is no consensus on the optimal secondary prevention strategy for ESUS patients. [32], [33] Two large randomized controlled trials did not demonstrate a benefit of direct oral anticoagulants in preventing recurrent strokes in ESUS patients, [34], [35] likely because up to 70 % of these patients do not have AF, and other sources of embolism may not respond to anticoagulation. Many of the other sources are arterial, which may explain why antiplatelet therapy was not inferior to anticoagulation. Our findings suggest that identifying patients unlikely to benefit from anticoagulation therapy is feasible. Specifically, ESUS patients with low D-dimer levels and unfavorable lipid profiles are less likely to benefit from anticoagulation, as their risk of AF is minimal. This approach may aid in optimizing secondary prevention strategies by focusing on tailored treatment decisions. Recent data suggest that statin therapy may reduce ischemic stroke recurrence in ESUS patients. [9] While our findings support the rationale for intensive dyslipidemia management in ESUS patients with unfavorable lipid profiles and low D-dimer levels, the specific role of statin use in this context was not directly evaluated in our study. Further prospective studies are needed to confirm the effectiveness of statin therapy in reducing recurrent stroke risk in this population. Nevertheless, due to the relatively small sample size and retrospective design of our study, these findings should be interpreted with caution and warrant validation through larger, prospective studies to confirm their generalizability and clinical utility.

## 5 Limitations

Several limitations should be acknowledged:

1. The sample size of this study is modest, which may limit the generalizability of the findings and the robustness of conclusions drawn from the retrospective data.
2. The model was developed using a small cohort and involved testing numerous variables, raising concerns about multiple testing. Validation in an external cohort or a prospective study with pre-specified criteria is essential to confirm the model's applicability.
3. The exclusion of the CHA2DS2-VASc score, while statistically justified due to multicollinearity, could be considered a limitation. Its inclusion might have improved sensitivity. Notably, based on this model, approximately 7 % of patients with AF might not receive ILR implantation, underscoring the need for cautious interpretation of these findings.
4. Diagnostic testing protocols for ESUS varied among patients, which may introduce heterogeneity; however, no significant differences were observed between the AF Group and No AF Group regarding the rates of these tests.
5. The detection of AF via ILR does not definitively confirm a cardioembolic stroke etiology, particularly for patients whose AF was detected after a prolonged period post-ILR implantation.
6. Some patients in the No AF Group may have underlying left atrial dysfunction (atrial cardiomyopathy) without overt AF, a limitation shared by similar studies.
7. The etiology of embolism in the No AF Group is presumed to be arterial, but there may be variability in the site and nature of the emboli, including contributions from non-atherosclerotic sources.

## 6 Conclusion

Combining D-dimer and LDL/HDL ratio provides an effective and accessible method for predicting the absence of AF in ESUS patients undergoing ILR monitoring. Here, 'absence of AF' refers specifically to patients in whom AF was not detected during the monitoring period, helping to identify those less likely to benefit from ILR use. The implementation of this predictive model could potentially optimize patient selection for ILR implantation, enhance secondary prevention strategies, and improve overall healthcare resource utilization.

**Funding Sources**.

None

**Ethics Declarations**

This study protocol was approved by the Ethics Committee of 10.13039/100019268Showa University, Japan, and was conducted in accordance with the principles of the Declaration of Helsinki (Approval Number: 3138). The requirement for informed consent was waived due to the retrospective nature of the study and the minimal risk to participants.

## CRediT authorship contribution statement

**Kosuke Yoshikawa:** Writing – original draft, Investigation, Formal analysis, Data curation. **Taku Asano:** Supervision. **Yoshimi Onishi:** Writing – review & editing, Writing – original draft, Visualization, Validation, Software, Resources, Project administration, Methodology, Investigation, Formal analysis, Data curation, Conceptualization. **Yuki Takai:** Writing – review & editing. **Hiroto Sugiyama:** Writing – review & editing. **Shuhei Arai:** Data curation. **Toshihiko Gokan:** Data curation. **Yuya Nakamura:** Data curation. **Koichiro Inokuchi:** Data curation. **Miwa Kikuchi:** Data curation. **Tatsuya Onuki:** Data curation. **Hitoshi Ezumi:** Data curation. **Shinji Koba:** Supervision. **Kaoru Tanno:** Data curation. **Youichi Kobayashi:** Supervision. **Toshiro Shinke:** Supervision.

## Declaration of competing interest

The authors declare that they have no known competing financial interests or personal relationships that could have appeared to influence the work reported in this paper.
